# A Systematic Review and Meta-Analysis of the Impact of Cancer and Its Treatment Protocol on the Success of Orthodontic Treatment

**DOI:** 10.3390/cancers15225387

**Published:** 2023-11-13

**Authors:** Mohammad Khursheed Alam, Mohammed Awawdeh, Sanjeev B. Khanagar, Wael Aboelmaaty, Huda Abutayyem, Haytham Jamil Alswairki, Ahmed Ali Alfawzan, Mohammad Younis Hajeer

**Affiliations:** 1 Orthodontic Division, Preventive Dentistry Department, College of Dentistry, Jouf University, Sakaka 72345, Saudi Arabia; 2 Department of Dental Research Cell, Saveetha Institute of Medical and Technical Sciences, Saveetha Dental College and Hospitals, Chennai 600077, India; 3 Department of Public Health, Faculty of Allied Health Sciences, Daffodil International University, Dhaka 1207, Bangladesh; 4 Preventive Dental Science Department, College of Dentistry, King Saud bin Abdulaziz University for Health Sciences (KSAU-HS), Riyadh 11426, Saudi Arabia; sanjeev.khanagar76@gmail.com; 5 King Abdullah International Medical Research Center, Ministry of National Guard—Health Affairs, Riyadh 11481, Saudi Arabia; wael_m_safwat@mans.edu.eg; 6 Dental Services King Abdulaziz Medical City, Ministry of National Guard—Health Affairs, Riyadh 11426, Saudi Arabia; 7 Visiting Associate Professor, College of Medicine & Dentistry, Ulster University, Birmingham B4 6BN, UK; 8 Maxillofacial Surgery and Diagnostic Sciences Department, College of Dentistry, King Saud bin Abdulaziz University for Health Sciences (KSAU-HS), Riyadh 11426, Saudi Arabia; 9 Oral Radiology and Diagnostic Sciences, Faculty of Dentistry, Mansoura University, Mansoura City 35516, Egypt; 10 Department of Clinical Sciences, Center of Medical and Bio-Allied Health Sciences Research, College of Dentistry, Ajman University, Ajman 346, United Arab Emirates; h.abutayyem@ajman.ac.ae; 11 School of Dental Sciences, Universiti Sains Malaysia, Kota Bharu 16150, Malaysia; hitham.swerki@gmail.com; 12 Department of Preventive Dentistry, College of Dentistry in Ar Rass, Qassim University, Ar Rass 52571, Saudi Arabia; ah.alfawzan@qu.edu.sa; 13 Department of Orthodontics, Faculty of Dentistry, University of Damascus, Damascus P.O. Box 16046, Syria; myhajeer@gmail.com

**Keywords:** cancer, chemotherapy, orthodontic treatment, aligner therapy, metastasis

## Abstract

**Simple Summary:**

This systematic review and meta-analysis examines the impact of cancer and its treatment on orthodontic treatment success. Existing studies show significant gaps in understanding the relationship between anti-cancer medications and orthodontic care, necessitating further research. The review selected five studies with varying methodologies, finding a strong association between radiotherapy, antineoplastic drugs, and reduced success in orthodontic treatment. The results indicate that while orthodontic treatments can be effective for children undergoing chemotherapy, their effectiveness may diminish in older populations. The review underscores the importance of considering cancer therapies in orthodontic planning to optimize results and minimize complications.

**Abstract:**

Background: There are several significant gaps in current studies of the relationship between anti-cancer medications and orthodontic care that call for more investigation. As a result, the main goals of this systematic review and meta-analysis were to summarise and assess the information that was available regarding the effect of radiotherapy and anti-cancer medications on the overall successful completion of an orthodontic treatment plan. Methods: A standardised data extraction form was devised in accordance with the PRISMA guidelines to conduct a systematic review and meta-analysis, with specific criteria implemented for selecting studies with low to moderate risk of bias. Results: Five studies involving different methodologies were selected at the conclusion of the search strategy. The statistical analysis revealed an estimated odds ratio (OR) of 0.31 and relative risk (RR) of 0.48, indicating a statistically significant association between the use of radiotherapy and anti-neoplastic drugs and a noticeable reduction in the successful completion of orthodontic treatment. The heterogeneity analysis showed significant heterogeneity among the studies. Conclusions: This review emphasises that, although orthodontic therapies can still be beneficial for children receiving chemotherapy, the effectiveness of the therapy may be diminished in older populations. The findings further highlight how crucial it is to take cancer therapies into account when planning and managing orthodontic treatment in order to optimise results and reduce problems.

## 1. Introduction

The role of an orthodontist in treating patients with occlusal or dental issues who are also suffering from systemic diseases is crucial for comprehensive and coordinated care [1]. Orthodontists play a significant role in evaluating and managing the dental and occlusal aspects of patients with systemic diseases, working in collaboration with other healthcare professionals to provide integrated treatment [2]. Firstly, orthodontists contribute their expertise in diagnosing and treating malocclusions and dental irregularities [3]. They assess the patient’s occlusion, tooth alignment, and jaw relationships to identify any specific orthodontic needs [3]. By analysing dental and facial structures, orthodontists can develop individualised treatment plans to address the patient’s occlusal issues, aiming to improve function, aesthetics, and overall oral health [4]. In the context of systemic diseases, orthodontists collaborate with other healthcare providers, such as primary care physicians, specialists, and medical teams involved in managing the patient’s systemic condition [5]. This interdisciplinary approach allows for a comprehensive understanding of the patient’s overall health status and ensures that orthodontic treatment aligns with the overall medical management plan [6]. Orthodontists also consider the impact of systemic diseases and their treatments on dental health [7]. Certain systemic conditions, such as diabetes, cardiovascular diseases, or autoimmune disorders, can affect oral health, including gum health, tooth mobility, and bone density [7].

Systemic diseases, such as cancer, can have profound effects on dental and oral health across different age groups [8]. The impact of cancer on dental and oral health can vary depending on factors such as the type and stage of cancer, the treatment protocols used, and individual characteristics [9]. For example, in cases of children and adolescents, certain cancers and cancer treatments can disrupt the normal progression of dental development, leading to delayed eruption of teeth or abnormalities in tooth morphology [10]. Chemotherapy or radiation therapy administered during childhood can also interfere with enamel formation, resulting in enamel hypoplasia or increased susceptibility to tooth decay [11]. In cases of adults, several conditions such as oral mucositis, xerostomia, and jaw osteonecrosis can occur as a result of medications prescribed to cancer patients [12]. Anti-cancer treatments also tend to weaken the immune system, making individuals more vulnerable to oral infections, such as fungal or viral infections like oral thrush or herpes simplex virus infection [12].

The existing literature exploring the correlation between anti-cancer therapies and orthodontic treatment reveals several notable gaps that highlight areas where further research is needed [13,14]. One significant gap is the lack of standardised protocols for orthodontic treatment in patients undergoing anti-cancer therapies. The absence of consistent guidelines hampers the ability to compare results across studies and develop evidence-based practices. Establishing standardised protocols would ensure uniformity and enable better assessment of treatment outcomes, aiding in the development of guidelines tailored to the specific needs of these patients. Furthermore, the limited sample sizes and lack of diversity in participant populations in existing studies pose limitations. The absence of consensus on outcome measures hinders effective comparison of results across studies and impedes the ability to conduct meaningful meta-analyses. Establishing agreed-upon outcome measures would facilitate the synthesis of findings and contribute to a more comprehensive understanding of the subject. Therefore, the primary objectives of this systematic review and meta-analysis were to synthesise and analyse the available evidence pertaining to the impact of radiotherapy and anti-neoplastic drugs on the overall successful completion of an orthodontic treatment plan.

## 2. Materials and Methods

### 2.1. Review Protocol

**Figure 1:**
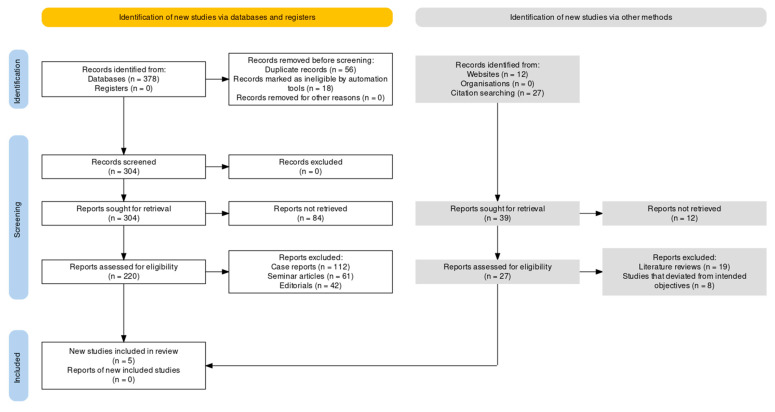
Article selection framework using the PRISMA guidelines.

The PRISMA (Preferred Reporting Items for Systematic Reviews and Meta-Analyses) strategy (shown in Figure 1) for this investigation was formulated and executed according to the PRISMA guidelines [15,16].

### 2.2. Review Design

The PICOS strategy (Population, Intervention, Comparison, Outcome, Study Design) for this systematic review and meta-analysis was formulated as follows:

Population: The target population consisted of individuals with occlusal or dental issues who were suffering from, or had recovered from, different types of cancer/neoplasms without any limitations on the age range or sex.

Intervention: The intervention of interest in this study was orthodontic treatment. This encompassed various orthodontic procedures aimed at addressing the occlusal or dental issues experienced by individuals who were undergoing or had recently undergone oncological treatment without any limitations to the type of orthodontic interventions or techniques employed across the included studies.

Comparison: The comparison in this study involved assessing the impact of cancer and its associated treatment regimen on dental and oral health in individuals with occlusal or dental issues. This typically involved comparing the oral health outcomes and treatment efficacy between individuals with cancer/neoplastic development and those without systemic diseases (i.e., healthy controls) without limiting the specific type of comparison.

Outcome: The primary outcomes of interest were the impact of cancer and its associated treatment regimen on dental and oral health. This encompassed various aspects, such as dental development, tooth enamel defects, malocclusions, oral mucositis, xerostomia, different classes of malocclusion, and other relevant parameters.

Study Design: The eligible study designs for inclusion in this systematic review and meta-analysis were retrospective studies, case-control studies, prospective case-control studies, and cross-sectional studies.

### 2.3. Search Protocol

The search strategy for this systematic review and meta-analysis of the impact of cancer and its treatment protocol on the success of orthodontic treatment involved searching six different online databases. The search strategy utilised MeSH (Medical Subject Headings) keywords and Boolean operators to ensure a comprehensive and focused search. The search strategy began by identifying the main concepts of the research question: cancer, orthodontic treatment, and treatment success. MeSH terms and keywords related to each concept were identified. For cancer, terms such as “neoplasms”, “cancer”, and specific cancer types (e.g., leukaemia and lymphoma) were included. For orthodontic treatment, terms such as “orthodontics”, “orthodontic procedures”, and “dental occlusion” were used. And for treatment success, terms like “treatment outcome”, “treatment efficacy”, and “success rate” were incorporated. The protocol is presented in Table 1.

### 2.4. Article Selection Criteria

The inclusion and exclusion criteria for this review were established to ensure the selection of relevant and high-quality studies. The criteria were defined based on the research question and the specific objectives of the review. Only primary research studies, including randomised controlled trials (RCTs), cohort studies, case-control studies, and cross-sectional studies, were considered for inclusion. The population of interest encompassed patients of any age group who were diagnosed with cancer and underwent orthodontic treatment. Various types of cancer, such as leukaemia, lymphoma, or solid tumours, were included. The review focused on studies investigating the impact of cancer and its treatment protocol on the success of orthodontic treatment, including the evaluation of orthodontic procedures, treatment outcomes, and measures of treatment success. Studies with a control or comparison group, such as healthy individuals without cancer or cancer patients receiving different treatment protocols, were included for comparative analyses. Relevant outcome measures related to orthodontic treatment success, including dental occlusion, malocclusion classification, treatment outcomes, or any other clinically relevant measure, were considered for inclusion. On the other hand, animal studies, review articles, case reports, non-English studies, and studies published before a specified cut-off date were excluded. Additionally, studies with insufficient data or incomplete reporting of outcomes that hindered data extraction or meta-analysis were excluded.

The chosen studies for review, however, included a thorough explanation of why the patients required orthodontic treatment and how the risks (such as periodontal infection, caries, and root resorption) were assessed and minimised in each study, given the population’s vulnerability owing to a potentially weakened immune system. It was also critical to assess if orthodontic treatment made the patients’ condition worse. It would have been extremely concerning and the studies might not have been appropriate for inclusion in our evaluation if the research that we analysed did not offer sufficient detail in these areas. As a result, we made sure that these factors were adequately taken into consideration while choosing the research for our review. We chose papers in which the authors discussed explicitly risk assessment and mitigation techniques as well as the reasoning behind orthodontic therapy in the particular demographic. Additionally, we made sure that any possibility of the patients’ condition getting worse as a result of orthodontic therapy was assessed. A study was excluded from our review if it did not sufficiently address these issues.

### 2.5. Data Extraction Protocol

The data extraction protocol for this study was carefully developed to ensure the systematic collection and organisation of relevant data from the selected studies. The protocol aimed to extract key information essential for addressing the research objectives and conducting a comprehensive analysis. The data extraction process involved multiple steps. Firstly, a standardised data extraction form was created, specifying the information to be extracted from each included study. The form included fields such as study characteristics (e.g., author, year of publication, and study design), participant characteristics (e.g., age and type of cancer), details of the orthodontic treatment (e.g., type of treatment and duration), and outcomes related to treatment success (e.g., occlusal outcomes, malocclusion classification, and patient-reported outcomes). To ensure accuracy and reliability, two independent reviewers conducted the data extraction process. They carefully reviewed each included study and extracted the relevant data using the predefined data extraction form. Any discrepancies or disagreements between the reviewers were resolved through discussion and consensus. In cases where a consensus could not be reached, a third reviewer was consulted to make a final decision. During the data extraction process, efforts were made to minimise errors and biases. The reviewers cross-checked their extracted data to ensure consistency and accuracy. Additionally, the extracted data were carefully entered into a standardised database or spreadsheet for further analysis. By following this rigorous data extraction protocol, the systematic review and meta-analysis aimed to ensure the systematic collection and accurate representation of data from the included studies, thereby providing a solid foundation for the subsequent analysis and synthesis of findings.

### 2.6. Evaluation of Bias

**Figure 2:**
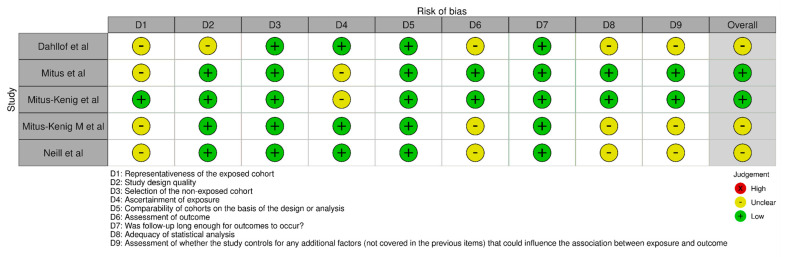
Intra-review bias assessment using NOS [19,20,21,22,23].

The bias assessment for this review was performed using the Newcastle-Ottawa Scale (NOS) tool (https://www.ohri.ca/programs/clinical_epidemiology/oxford.asp (accessed on 6 November 2023)) [17,18]. The NOS tool (Figure 2) is commonly used to assess the quality and risk of bias in non-randomised studies, such as cohort studies and case-control studies, which were included in this review. The reviewers evaluated the included studies using the NOS tool to assess the risk of bias in three domains: selection of participants, comparability of groups, and ascertainment of outcomes. Each domain consists of specific criteria that are assigned a score. The reviewers carefully examined each study and assigned a score based on the fulfilment of these criteria.

### 2.7. Statistical Protocol

The meta-analysis protocol for this study, specifically focusing on the impact of radiotherapy and anti-neoplastic drugs on the overall successful completion of an orthodontic treatment plan, was conducted using RevMan 5 (version 5.4.1).

The analysis was performed assuming a random-effects model, which accounts for the expected heterogeneity across the studies included in the meta-analysis. The 95% confidence interval (CI) was used to estimate the precision of the treatment effect and to determine the statistical significance of the findings. To begin the analysis, the relevant data extracted from the included studies were entered into the RevMan software. The primary outcome measure, which in this case was the overall successful completion of an orthodontic treatment plan, was selected for the meta-analysis. The data from the individual studies were then pooled together to calculate the overall effect size, expressed as an odds ratio (OR) and risk ratio (RR) with a corresponding 95% CI. The forest plots were generated to visually display the results of the meta-analysis, with each study represented by a point estimate and a confidence interval. In addition to the forest plot, statistical measures of heterogeneity were assessed to determine the consistency of the results across the included studies. This included calculating the chi-square test statistic and the I² statistic, which quantifies the percentage of total variation across studies due to heterogeneity rather than chance.

## 3. Results

Table 2 presents a summary of the included studies without delving into the specific details of each study. The studies were conducted in different regions and spanned various years. Dahllof et al. [19] conducted their study in Sweden with a relatively small sample size of 10 participants, all of whom were under the age of 12. The study included six female participants, but no information was provided about the sex ratio beyond that. Mitus et al. [20] conducted their study in Poland and had a larger sample size of 104 participants, with a mean age of 19.6 years. The majority of the participants in this study were female, with 46 included. However, the sex ratio beyond that was not provided. Mitus–Kenig et al. [21] also conducted their study in Poland, with a sample size of 80 participants. The mean age of the participants was not specified in this study. Out of the 80 participants, 17 were females, but no additional information about the sex ratio was given. In another study by Mitus–Kenig et al. [22] in Poland, again with a sample size of 80 participants, the mean age was reported as 19.3 years. The majority of participants in this study were female, with 52 included. However, similar to the previous studies, the exact sex ratio was not provided. Lastly, Neill et al. [23] conducted a study in the USA with a larger sample size of 381 participants. The mean age and sex ratio for this study were unspecified.

The findings from Table 3 provide valuable insights into the impact of cancer treatments on orthodontic outcomes, specifically in relation to different malocclusions and orthodontic issues. All selected papers reported on patients who were either cancer survivors [19,20,22,23] or in the post-cancer therapy stage [21]. One study [19] involved a retrospective analysis with a long 20-year follow-up period, focusing on children who underwent bone marrow transplantation due to leukaemia. Despite receiving chemotherapy, 60% of the children achieved ideal orthodontic treatment results, indicating that orthodontic interventions can still be effective in this population. A case-control study [20] compared cancer patients to healthy controls and evaluated Class I, II, and III malocclusions, primarily in leukaemia patients. Over a three-year follow-up period, the research demonstrated that chemotherapy significantly reduced the efficacy of the administered orthodontic treatment, implying a negative impact on orthodontic outcomes. Another case-control study [21] included cancer patients with leukaemia and brain tumours, comparing them to healthy controls. The four-year assessment period revealed that oncological patients’ treatment outcomes following properly executed orthodontic procedures did not differ from those of healthy controls, indicating that the orthodontic outcomes were similar in both groups. In a prospective case-control study [22], patients with leukaemia, neuroblastoma, and non-Hodgkin’s lymphoma were compared to healthy controls. The study spanned seven years and found that cancer patients experienced a significant decline in quality of life prior to the start of orthodontic therapy. However, there was a significant improvement in quality of life after treatment, suggesting that the outcomes of orthodontic treatment for cancer survivors were comparable to those of healthy controls. A cross-sectional study [23] examined developmental disturbances and orthodontic issues in patients who received different forms of cancer treatment. The results showed that radiation therapy, either alone or in combination with other treatments, was five times more likely to result in root resorption or microdontia compared to chemotherapy or any other form of treatment. Conversely, patients who only received chemotherapy were almost three times more likely to have no dental complications compared to those who received other forms of treatment. The findings indicate that cancer treatments can have varying effects on orthodontic outcomes. While orthodontic treatment can still produce ideal results in children undergoing chemotherapy, the efficacy of orthodontic interventions may be reduced by chemotherapy. However, properly executed orthodontic procedures can yield comparable treatment outcomes between cancer patients and healthy individuals. Additionally, the type of cancer treatment plays a role in dental complications, with patients undergoing radiation therapy having a higher likelihood of root resorption or microdontia compared to those administered chemotherapy. These findings highlight the importance of considering cancer treatments in orthodontic planning and management to optimise treatment outcomes and minimise complications.

**Figure 3:**
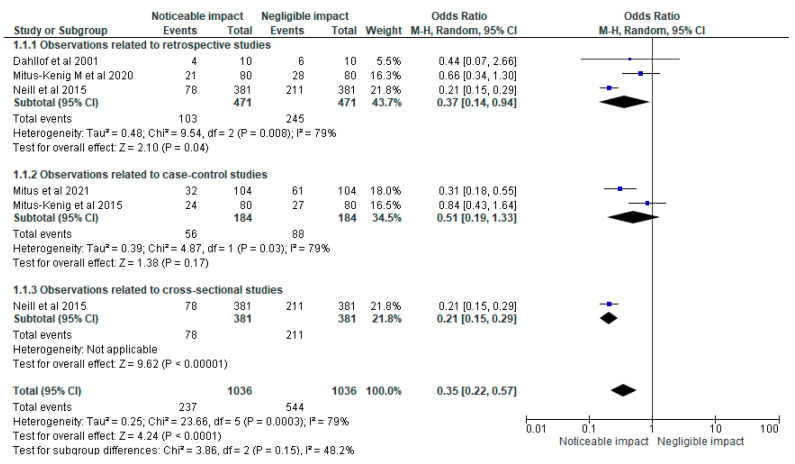
Impact of radiotherapy and anti-neoplastic drugs on the overall successful completion of an orthodontic treatment plan represented in terms of the OR [19,20,21,22,23].

The forest plot shown in Figure 3 presents a comprehensive analysis of the impact of radiotherapy and anti-neoplastic drugs on the overall successful completion of an orthodontic treatment plan. The odds ratio (OR) was estimated at 0.31, with a 95% confidence interval (CI) ranging from 0.25 to 0.40. This indicates that there was a statistically significant association between the use of radiotherapy and anti-neoplastic drugs and a noticeable reduction in the successful completion of orthodontic treatment. The forest plot also provided information regarding the heterogeneity of the studies included in the analysis. The chi-square test for heterogeneity yielded a value of 19.63, with four degrees of freedom (df), resulting in a *p*-value of 0.0006. This suggests that there was significant heterogeneity among the studies. Furthermore, the I-squared statistic was calculated to be 80%, indicating substantial heterogeneity among the included studies. This suggests that the variation in the effect sizes observed across the studies was not solely due to chance, but rather reflects real differences in the impact of radiotherapy and anti-neoplastic drugs on orthodontic treatment outcomes. Lastly, the test for the overall effect demonstrated a significant association between radiotherapy, anti-neoplastic drugs, and the overall success of orthodontic treatment. The Z-statistic was calculated to be 9.71, with a *p*-value of less than 0.00001. This provides strong evidence in favour of the hypothesis that radiotherapy and anti-neoplastic drugs have a significant impact on the successful completion of orthodontic treatment plans.

**Figure 4:**
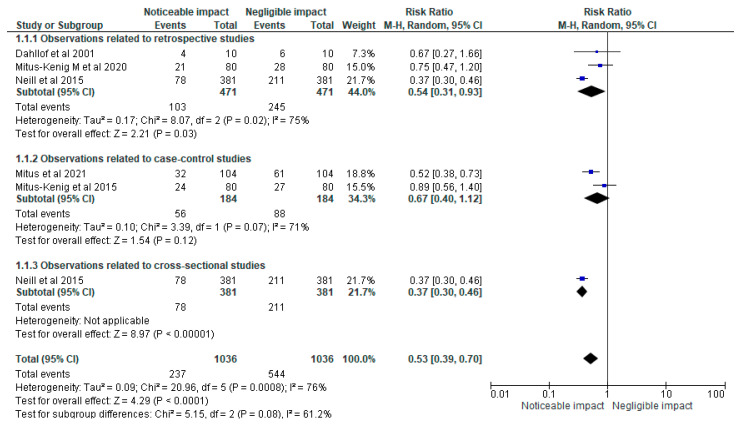
Impact of radiotherapy and anti-neoplastic drugs on the overall successful completion of an orthodontic treatment plan represented in terms of the RR [19,20,21,22,23].

Figure 4’s forest plot shows a comprehensive statistical analysis examining the impact of radiotherapy and anti-neoplastic drugs on the overall successful completion of an orthodontic treatment plan. The analysis revealed an estimated relative risk (RR) of 0.48, with a 95% confidence interval (CI) ranging from 0.41 to 0.56. This indicates a significant association between the use of radiotherapy and anti-neoplastic drugs and a noticeable reduction in the successful completion of orthodontic treatment. Additionally, the forest plot included information on the heterogeneity of the included studies. The chi-square test for heterogeneity yielded a value of 16.83, with four degrees of freedom (df), resulting in a *p*-value of 0.002. This suggests that there was significant heterogeneity among the studies, indicating potential variations in study design, patient populations, or other factors contributing to the observed differences in treatment outcomes. Moreover, the I-squared statistic was calculated to be 76%, indicating a substantial level of heterogeneity among the included studies. This suggests that a considerable portion of the variability in effect sizes across the studies is not due to chance alone but rather reflects genuine differences in the impact of radiotherapy and anti-neoplastic drugs on orthodontic treatment outcomes. Finally, the test for the overall effect demonstrated a significant association between radiotherapy, anti-neoplastic drugs, and the overall success of orthodontic treatment. The Z-statistic was calculated to be 9.33, with a *p*-value of less than 0.00001. These results provide strong evidence supporting the hypothesis that radiotherapy and anti-neoplastic drugs have a substantial impact on the successful completion of orthodontic treatment plans.

## 4. Discussion

This review provides significant insights into the correlation between cancer treatments and orthodontic outcomes. The findings suggest that orthodontic interventions can still be effective in children undergoing chemotherapy, with 60% achieving ideal treatment results. However, chemotherapy was found to reduce the efficacy of orthodontic treatment for certain malocclusions. This study also revealed that the type of cancer treatment plays a role in dental complications, with radiation therapy increasing the likelihood of root resorption or microdontia compared to chemotherapy. These findings have important implications for orthodontic planning and management in cancer patients, highlighting the need to consider cancer treatments to optimise outcomes and minimise complications. This study is unique and insightful in its findings, as it presents a comprehensive analysis using a forest plot to assess the impact of radiotherapy and anti-neoplastic drugs on the overall success of orthodontic treatment. The analysis showed a statistically significant association between the use of radiotherapy and anti-neoplastic drugs and a noticeable reduction in the successful completion of orthodontic treatment. The heterogeneity analysis indicated significant variation among the included studies, suggesting potential differences in study design and patient populations. The overall effect test further confirmed the significant association between radiotherapy, anti-neoplastic drugs, and the successful completion of orthodontic treatment.

Orthodontists tend to face numerous challenges when treating patients with occlusal or dental issues who also suffer from systemic diseases [24]. These challenges stem from the complex interplay between the systemic condition, its treatments, and the goals of orthodontic treatment [25]. Key challenges include carefully considering the patient’s medical history and coordinating with their healthcare team to ensure safe and effective orthodontic care. The timing and duration of treatment may need to be adjusted based on the systemic disease and its treatments [26]. Oral complications resulting from systemic conditions, such as mucositis or compromised wound healing, must be managed in collaboration with the medical team [27]. Interactions between medications used in systemic disease treatment and orthodontic treatment need to be taken into account. Maintaining proper oral hygiene can be challenging for patients with compromised immunity or physical limitations, requiring guidance and support from orthodontists [28]. Multidisciplinary collaboration among healthcare professionals is crucial to align treatment goals and manage potential complications [29]. Considering the emotional and psychological impact of the systemic disease on patients and providing appropriate support throughout the treatment process is essential for their comfort and well-being [30].

One of the fundamental issues in modern orthodontics is the long-term stability of the orthodontic treatment. According to one study [31], only 7.1% of cases with long-term stability (between four and 10 years following the conclusion of orthodontic treatment) could be classified as presenting absolute stability, while 68.6% of cases were classified as presenting relative stability. The scientists found that overbite and lower anterior segment alignment were the least persistent occlusal characteristics. Another study [32] examined the causes of relapse following orthodontic therapy. They cited a number of variables that affect the stability of orthodontic treatment, such as periodontal and gingival variables, occlusal variables, soft tissue pressures, dentition limits, and variables that cause so-called physiological relapse. Additionally, a different study [33] focused on the significance of the mandibular muscles in maintaining long-term occlusal stability. The majority of cancer survivors, according to the literature, had at least one type of dental complication, such as misaligned teeth, root stunting, changes in growth and development, missing teeth, a delay in the loss of deciduous teeth, microdontia, and enamel hypoplasia [23,34,35]. Additionally, it was discovered that chemotherapy was associated with early apexification, stunted root growth, and tooth discolouration as well as poorer oral hygiene, higher caries intensity, oral lesions, and hyposalivation [34]. The development of the tooth’s crown and roots are affected by both chemotherapy and radiotherapy, with root abnormalities being more common [35]. Impaired root growth was the most frequent root defect, whereas microdontia was the most frequent crown fault [35]. Additionally, because both chemotherapy and radiation are utilised in contemporary oncological treatment modalities, it is impossible to discern between the problems in odontogenesis produced by either one alone [35,36,37].

This review has several limitations that should be considered. Firstly, the findings were based on a limited number of studies, and the sample sizes within each study varied. This variation in sample sizes could introduce bias and affect the generalisability of the results. Secondly, the studies included in this review predominantly focused on a limited number of cancer types, such as leukaemia and brain tumours, which may limit the generalisability of the findings to other cancer populations. Additionally, this review primarily relied on retrospective and case-control studies, which are susceptible to recall bias and may not establish causality. Prospective studies with larger sample sizes and longer follow-up periods would provide more robust evidence. Moreover, the heterogeneity analysis revealed significant variation among the included studies, suggesting potential differences in study design, patient populations, or other factors that may influence treatment outcomes. This heterogeneity introduces uncertainty and calls for a cautious interpretation of the results. Lastly, while the forest plots demonstrated statistically significant associations between radiotherapy, anti-neoplastic drugs, and the success of orthodontic treatment, the observed effect sizes may still be subject to confounding factors that were not accounted for in the analysis. Further research considering potential confounders and addressing the limitations mentioned would enhance the understanding of the impact of cancer treatments on orthodontic outcomes.

### Recommendations for Clinical Applications

The impact of cancer treatments on orthodontic outcomes necessitates individualised treatment planning. A significant proportion of children can achieve ideal orthodontic treatment results despite undergoing chemotherapy, underscoring the efficacy of orthodontic interventions among this population. However, chemotherapy may reduce the efficacy of orthodontic treatment in certain cases, implying a possible negative impact on orthodontic outcomes. Orthodontic outcomes for cancer patients, when treatment procedures are properly executed, can be on par with those of healthy individuals. This highlights the importance of meticulous planning and execution in orthodontic procedures. Quality of life, which may decline significantly before the start of orthodontic therapy in cancer patients, improves markedly post-treatment. This suggests that orthodontic treatment can contribute positively to the well-being of cancer survivors. The type of cancer treatment plays a crucial role in dental complications. Radiation therapy, either alone or in combination with other treatments, is more likely to result in root resorption or microdontia compared to chemotherapy. Conversely, chemotherapy alone is less likely to cause dental complications. Statistical analyses reaffirm the influence of cancer treatments, particularly radiotherapy and anti-neoplastic drugs, on orthodontic treatment outcomes. Both analyses show a significant association between these treatments and a reduced success rate of orthodontic treatment. These findings highlight the need for integrating the knowledge of cancer treatments into orthodontic planning and management to optimise outcomes and minimise complications.

## 5. Conclusions

This review highlights that, while orthodontic interventions can still be effective in children undergoing chemotherapy, the efficacy of treatment may be reduced when taking into account individuals of advanced ages. However, properly executed orthodontic procedures can yield comparable outcomes between cancer patients and healthy individuals. The type of cancer treatment also plays a role, with radiation therapy having a higher likelihood of dental complications compared to chemotherapy. The findings emphasise the importance of considering cancer treatments in orthodontic planning and management to optimise outcomes and minimise complications. Further research with larger sample sizes and prospective designs is needed to strengthen the evidence and address potential confounders.

## Figures and Tables

**Table 1:** Search protocol across databases.

Database	Search Strategy
PubMed	(((neoplasms[MeSH Terms] OR cancer) AND (orthodontics[MeSH Terms] OR orthodontic procedures) AND (treatment outcome[MeSH Terms] OR treatment efficacy OR success rate)))
Embase	(((neoplasms/OR cancer) AND (orthodontics/OR orthodontic procedures) AND (treatment outcome/OR treatment efficacy OR success rate)))
Cochrane Library	(((neoplasms[MeSH Terms] OR cancer) AND (orthodontics[MeSH Terms] OR orthodontic procedures) AND (treatment outcome[MeSH Terms] OR treatment efficacy OR success rate)))
Web of Science	(((neoplasms OR cancer) AND (orthodontics OR orthodontic procedures) AND (treatment outcome OR treatment efficacy OR success rate)))
Scopus	(((neoplasms OR cancer) AND (orthodontics OR orthodontic procedures) AND (treatment outcome OR treatment efficacy OR success rate)))
CINAHL	(((neoplasms OR cancer) AND (orthodontics OR orthodontic procedures) AND (treatment outcome OR treatment efficacy OR success rate)))

**Table 2:** Demographic factors assessed in the included studies.

Author	Year	Region	Sample Size (n)	Mean Age (in Years)	Sex Ratio
Dahllof et al. [19]	2001	Sweden	10	<12	6 females
Mitus et al. [20]	2021	Poland	104	19.6	46 females
Mitus–Kenig et al. [21]	2015	Poland	80	Unspecified	17 females
Mitus–Kenig M et al. [22]	2020	Poland	80	19.3	52 females
Neill et al. [23]	2015	USA	381	Unspecified	Unspecified

**Table 3:** Oncological variables and assessments observed in the included studies.

Author	Protocol	Malocclusion/Orthodontic Issues Assessed	Type of Cancer Assessed	Follow-Up Period	Results Observed
Dahllof et al. [19]	Retrospective	Developmental disturbances such as microdontia, enamel hypoplasia, and other issues such as crowding and overjet	Chronic myeloid leukaemia, Gaucher’s disease, Acute myeloid leukaemia, Acute lymphoblastic leukaemia, T-cell acute lymphoblastic leukaemia, Acute myeloid leukaemia, Bruton’s disease, Fanconi’s anaemia	20 years (assessment period)	In 60% of the children, orthodontic treatment produced ideal results despite the administration of chemotherapy.
Mitus et al. [20]	Case-control (52 cancer patients and 52 healthy controls	Class I, II, and III malocclusions	Leukaemia (majority)	3 years	Chemotherapy significantly reduced efficacy of orthodontic treatment administered.
Mitus–Kenig et al. [21]	Case-control (40 cancer patients and 40 healthy controls)	Class I, II, and III malocclusions	Leukaemia and brain tumours	4 years (assessment period)	Oncological patients’ treatment outcomes following a properly executed orthodontic procedure did not differ from those of healthy controls.
Mitus–Kenig M et al. [22]	Prospective case-control (40 cancer patients and 40 healthy controls)	Class I, II, and III malocclusions	Leukaemia, neuroblastoma andnon-Hodgkin’s lymphoma	7 years (assessment period)	In the group of cancer patients, there was a significant decline in quality of life prior to the start of orthodontic therapy and a significant improvement after treatment, showing that the outcomes of orthodontic treatment for cancer survivors were the same as those for healthy controls.
Neill et al. [23]	Cross-sectional	Developmental disturbances such as microdontia, enamel hypoplasia, and other issues such as crowding and overjet	Unspecified	Unspecified	Radiation therapy alone or in combination with any other form of treatment was five times more likely to result in root resorption or microdontia than chemotherapy or any other form of treatment. Patients who had only received chemotherapy were almost three times more likely to have no dental complications than patients who received any other form of treatment.
